# The Development and Evaluation of a Text Message Program to Prevent Perceived Insufficient Milk Among First-Time Mothers: Retrospective Analysis of a Randomized Controlled Trial

**DOI:** 10.2196/17328

**Published:** 2020-04-29

**Authors:** Jill R Demirci, Brian Suffoletto, Jack Doman, Melissa Glasser, Judy C Chang, Susan M Sereika, Debra L Bogen

**Affiliations:** 1 Department of Health Promotion & Development University of Pittsburgh School of Nursing Pittsburgh, PA United States; 2 Department of Emergency Medicine University of Pittsburgh School of Medicine Pittsburgh, PA United States; 3 Office of Academic Computing Western Psychiatric Institute and Clinic University of Pittsburgh Pittsburgh, PA United States; 4 Department of Obstetrics, Gynecology & Reproductive Sciences, and Internal Medicine University of Pittsburgh School of Medicine Pittsburgh, PA United States; 5 Department of Health & Community Systems Center for Research and Evaluation University of Pittsburgh School of Nursing Pittsburgh, PA United States; 6 Department of Pediatrics Children’s Hospital of Pittsburgh University of Pittsburgh School of Medicine Pittsburgh, PA United States

**Keywords:** breast feeding, perceived insufficient milk, text messaging, short message service, cell phone, mobile phone, telemedicine, mHealth, randomized controlled trial

## Abstract

**Background:**

Several recent trials have examined the feasibility and efficacy of automated SMS *text messaging* to provide remote breastfeeding support to mothers, but these texting systems vary in terms of design features and outcomes examined.

**Objective:**

This study examined user engagement with and feedback on a theory-grounded SMS text messaging intervention intended to prevent perceived insufficient milk (PIM)—the single, leading modifiable cause of unintended breastfeeding reduction and cessation.

**Methods:**

We recruited 250 nulliparous individuals intending to breastfeed between 13 and 25 weeks of pregnancy in southwestern Pennsylvania. Participants were randomly assigned with equal allocation to either an SMS intervention to prevent PIM and unintended breastfeeding reduction or cessation (MILK, a Mobile, semiautomated text message–based Intervention to prevent perceived Low or insufficient milK supply; n=126) or a control group receiving general perinatal SMS text messaging–based support via the national, free Text4Baby system (n=124). Participants in both groups received SMS text messages 3 to 7 times per week from 25 weeks of pregnancy to 8 weeks postpartum. The MILK intervention incorporated several automated interactivity and personalization features (eg, keyword texting for more detailed information on topics and branched response logic) as well as an option to receive one-on-one assistance from an on-call study lactation consultant. We examined participant interactions with the MILK system, including response rates to SMS text messaging queries. We also sought participant feedback on MILK content, delivery preferences, and overall satisfaction with the system via interviews and a remote survey at 8 weeks postpartum.

**Results:**

Participants randomized to MILK (87/124, 70.2% white and 84/124, 67.7% college educated) reported that MILK texts increased their breastfeeding confidence and helped them persevere through breastfeeding problems. Of 124 participants, 9 (7.3%) elected to stop MILK messages, and 3 (2.4%) opted to reduce message frequency during the course of the study. There were 46 texts through the MILK system for individualized assistance from the study lactation consultant (25/46, 54% on weekends or after-hours). The most commonly texted keywords for more detailed information occurred during weeks 4 to 6 postpartum and addressed milk volume intake and breastfeeding and sleep patterns. MILK participants stated a preference for anticipatory guidance on potential breastfeeding issues and less content addressing the benefits of breastfeeding. Suggested improvements included extending messaging past 8 weeks, providing access to messaging for partners, and tailoring content based on participants’ pre-existing breastfeeding knowledge and unique breastfeeding trajectory.

**Conclusions:**

Prenatal and postpartum evidence–based breastfeeding support delivered via semiautomated SMS text messaging is a feasible and an acceptable intervention for first-time mothers. To optimize engagement with digital breastfeeding interventions, enhanced customization features should be considered.

**Trial Registration:**

ClinicalTrials.gov NCT02724969; https://clinicaltrials.gov/ct2/show/NCT02724969

## Introduction

Over 80% of mothers in the United States begin breastfeeding after their baby is born [1], indicating a broad awareness of its health and economic advantages [2,3]. However, only few mothers in the United States meet the national recommendations for breastfeeding duration (1+ years) and exclusivity (6 months) [4]. By 6 months, only 25% of mothers are breastfeeding without formula, and by 12 months, 36% are breastfeeding at all [1]. Only one-third of mothers reach their intended breastfeeding goals [5,6]. These data indicate that, although most mothers wish to breastfeed, they face significant barriers to doing so.

Perceived insufficient milk (PIM) is the single most common reason for formula supplementation and its corollary, premature breastfeeding cessation [5,7,8]. Approximately 35% of cases of weaning before intended breastfeeding duration are attributable to PIM [9]. Excluding those with adverse metabolic profiles (eg, diabetes and polycystic ovarian syndrome), PIM is rarely rooted in primary anatomical or physiological abnormalities [10,11]; rather, there is a strong relationship between PIM, low maternal breastfeeding self-efficacy, and misinformation about normal infant breastfeeding behavior, milk volume trajectories, and principles of milk production [12-14]. When left unchecked, these issues have the potential to propagate a ripple effect of formula supplementation, less frequent milk removal from the breast, and responsive physiological reductions in breast milk volume [9,15].

Few interventions exist to prevent or correct PIM and its consequences. One exception to this is a recent single group, pretest/posttest pilot study that examined a home-based education intervention to reduce PIM and increase maternal breastfeeding self-efficacy. The authors found that among 14 included mothers, self-efficacy increased and the attribution of infant crying to PIM decreased over time [16]. However, with no comparison group, it is difficult to separate the intervention from time effects. Moreover, the logistics of disseminating such an intervention are dependent on qualified personnel and resources, which are unavailable at many sites.

Automated SMS *text messaging* may be an effective platform to deliver targeted, evidence-based breastfeeding education and support to pregnant and lactating parents addressing the root causes of PIM. Mobile health interventions, most predominantly SMS text messaging–based interventions, to address global maternal-child health issues have proliferated in recent years. This trend reflects the ubiquity of cell phones and SMS text messaging, particularly among Generation X and Y women [17-19]. Several recent trials conducted both in developed and developing countries demonstrate significant increases in exclusive breastfeeding from 9 weeks to 6 months postpartum among women who received automated SMS text message breastfeeding support vs control group women [20-22]. However, to date, no published studies have examined SMS text messaging to address PIM specifically, and few have incorporated theory-based content and advanced functionality, such as automated interactivity and personalization, to engage breastfeeding mothers. In this study, we have reported on the usability and acceptability as well as design and implementation considerations of a semiautomated SMS text message system (ie, employing both automated responses as well as opportunities for live interactions) to prevent PIM, examined within the MILK (a Mobile, semiautomated text message–based Intervention to prevent perceived Low or insufficient milK supply) trial.

## Methods

### Design

MILK was a randomized controlled trial examining the effect of a theory-driven SMS text message breastfeeding support system vs an attention control condition (general perinatal text-based support from the national Text4Baby system) on PIM and breastfeeding outcomes among first-time mothers in the United States. We hypothesized that MILK would be feasible and, at 8 weeks postpartum, MILK participants would have a perception of greater breast milk volume/supply, higher self-reported breastfeeding confidence and satisfaction, lower anxiety related to breastfeeding, and higher rates of any and exclusive breastfeeding compared with the control group. Data collection was completed in May 2019, with breastfeeding outcome data pending (ClinicalTrials.gov registration: NCT02724969). This study reported on feasibility data only.

### Participants and Setting

Participants were recruited at prenatal visits during the second pregnancy trimester at University of Pittsburgh Medical Center Magee-Women’s Hospital prenatal clinics. Recruitment also occurred through local advertising (posted flyers and bus advertisements), a university research registry, and social media (TrialSpark). Eligible individuals were nulliparous, aged 18 years or older, English-speaking, 13 to 25 gestational weeks, pregnant with 1 infant, owned a cell phone with internet access (ie, a *smartphone*) and an unlimited SMS text message plan, and intended to exclusively or nearly exclusively breastfeed (<2 ounces of formula per day) for at least two months. Breastfeeding intention was assessed via a list of potential feeding options in the screener form; no information was provided for or against breastfeeding during the study introduction before screening or randomization. The exclusion criteria included any contraindications to breastfeeding [4] and maternal or fetal conditions likely to compromise breastfeeding or milk supply (eg, breast reduction surgery and major congenital anomalies). All participants provided written informed consent for study participation. The study was approved by the University of Pittsburgh Institutional Review Board.

### Procedures

After screening for eligibility and enrollment at 13 to 25 weeks of pregnancy, participants were randomized with equal allocation to the intervention or control group. Enrollment timing was based on both capturing a participant pool with viable pregnancies and maximizing between-group comparability in intervention timing (ie, commencement of breastfeeding-specific texts at 25 weeks for both groups). Control group participants received sign-up information for the freely available national Text4Baby program, which delivered automated texts immediately following enrollment; these texts included content on various aspects of infant care, including breastfeeding (seven prenatal breastfeeding-specific messages and three postpartum breastfeeding-specific messages before the primary study end point at 8 weeks postpartum). The Text4Baby program was developed by the National Healthy Moms, Healthy Babies Coalition in collaboration with the Centers for Disease Control and Prevention through a rigorous process including expert review, research, and input from pregnant and postpartum women. Text4Baby messages are continually updated in partnership with various public health organizations, and evidence suggests that the messages impact both maternal attitudes and health behaviors [23-25]. Participants assigned to MILK received messages beginning at 25 weeks of gestation that focused specifically on establishing breastfeeding confidence and behaviors to prevent PIM. All participants received prenatal and postpartum text messages 3 to 7 times per week. Although there was no postpartum cutoff point for Text4Baby messages, MILK texts continued till 8 weeks postpartum.

Baseline data, including demographics and health history, were collected via electronic medical record abstraction and maternal self-reporting at the enrollment visit. A remotely administered 8-week postpartum survey assessed participants’ overall enthusiasm for the MILK system and content preferences. MILK participants were additionally invited to participate in an audio-recorded phone or in-person interview about their study experiences at 8 weeks postpartum or at the time of breastfeeding cessation. Interviews were conducted by the study coordinator, followed a semistructured script, and were professionally transcribed.

### MILK Development

Development of the MILK intervention was based on the breastfeeding self-efficacy (social cognitive theory) conceptual model that theorizes self-efficacy as a driving force of breastfeeding behavior and PIM as a consequence of impaired breastfeeding self-efficacy [26,27]. In this framework, text content targeted antecedents of self-efficacy, including performance accomplishments (eg, development of early technical, hands-on breastfeeding skills), vicarious experiences (eg, video- and vignette-based breastfeeding exposure), verbal persuasion (eg, information on breastfeeding benefits), and physiological and affective states (eg, strategies to reduce anxiety around breastfeeding and milk production). Text content and delivery strategies were also informed by (1) principles of health communication and behavioral economics, (2) mapping of the breastfeeding trajectory and problems encountered by primiparous mothers in an ecological momentary assessment (EMA) study [28], (3) consultation with experts in human lactation and social marketing (Best for Babes Foundation), and (4) a focus group with pregnant and postpartum individuals to obtain feedback on draft messages.

The final text bank contained a series of 63 messages to be delivered from 25 to 40 weeks of pregnancy, and a series of 47 messages to be delivered from the day of delivery to 8 weeks postpartum. Content differed by the perinatal stage—antenatal messaging focused on positive reinforcement regarding the decision to breastfeed, the impact of breastfeeding on maternal and child health, current breastfeeding recommendations and goal setting, lactation-related body changes during pregnancy, and anticipatory guidance for how breastfeeding looks and works. In the postpartum period, texts encompassed anticipatory guidance about breastfeeding milestones and infant breastfeeding behavior, milk volume expectations, technical aspects of breastfeeding and problem resolution (eg, positioning and latch), referrals to local and online breastfeeding resources, and encouragement to begin and continue breastfeeding.

### MILK Platform and Design Features

The MILK text message system was built by investigators using an encrypted Structured Query Language server database at the University of Pittsburgh, with all outgoing texts and incoming participant responses managed through a Microsoft Access front-end server. The server stored all prespecified libraries of automated messages and contingent responses. Messages were typically scheduled to be delivered at 10 AM, with those longer than 160 characters split into several separate SMS text messages. The server additionally sent the study staff email notifications of participant replies and allowed the exchange of nonautomated texts between participants and study personnel. To ensure timely transition from prenatal to postpartum messages, participants were sent SMS reminders in the third trimester to text the keyword BIRTH after delivery; hospital delivery records were also checked daily to manually drive this transition if necessary.

MILK texts included free-standing content as well as embedded links to Web pages, infographics, photos, and videos. Texts also featured automated personalization and interactivity, such that mothers and their infants were addressed by name, the content was tailored to the infant’s gestational and chronological age, and participants could text keywords to receive more detailed topical information by email or text (37 texts included keyword prompts). A portion of texts also attempted to engage mothers by requesting a response and using branched logic algorithms to respond in kind. For example, in one text message series, participants were queried about the number of breastfeeding sessions in the last 24 hours, and automated responses provided feedback on whether the frequency was considered adequate, along with potential recourses if it was not. Additional features of the MILK system included the ability for participants to text a key phrase to stop messages or reduce message frequency to one series per week. Participants could also text HELP to communicate directly with a study-based international board-certified lactation consultant (IBCLC) via an SMS text message, an email, or a telephone call.

### Analysis

MILK metadata addressing interactions with the system were abstracted from the Access database. Summary statistics were calculated for quantitative survey data using IBM SPSS Statistics version 24 (IBM Corporation, Armonk, New York, 2016). We used an editing approach for the qualitative coding within the qualitative coding program ATLAS.ti (ATLAS.ti GmbH, Berlin, 2019) [29], adding and refining codes in an iterative manner. A total of 2 authors trained in qualitative methods coded each interview transcript independently and met to compare the coding and organize codes into major and minor themes. Any discrepancies in interpretation were to be adjudicated by a third investigator; and none occurred.

## Results

### Recruitment and Sample Characteristics

**Figure 1:**
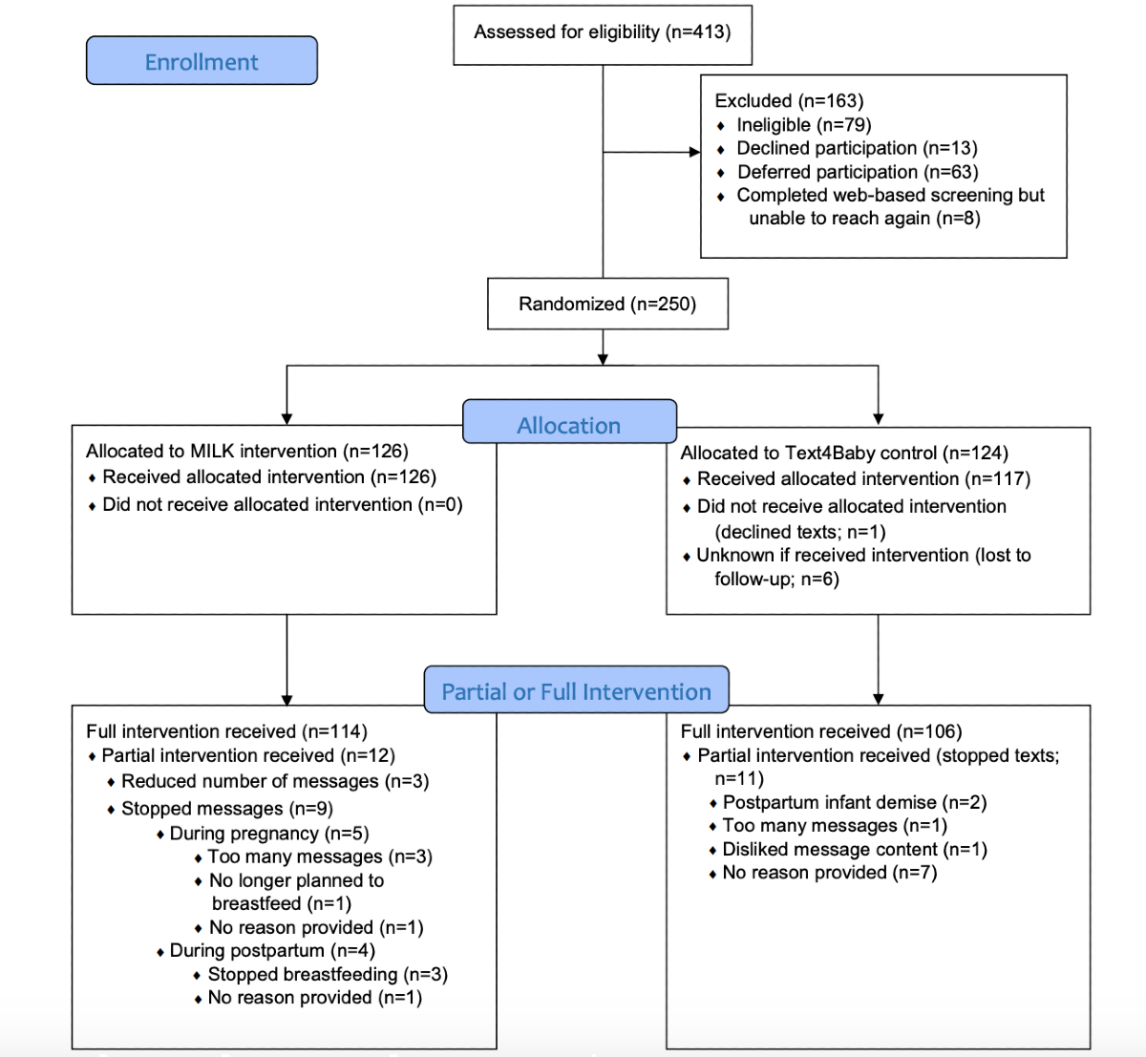
Study flow diagram. MILK: a Mobile, semiautomated text message–based Intervention to prevent perceived Low or insufficient milK supply.

A total of 250 maternal participants were recruited and randomized over a 15-month period (n=126 for MILK; n=124 for Text4Baby). The majority of the sample was recruited at prenatal visits (198/250, 79.2%). Among those assessed for eligibility, 3.2% (8/250) declined participation and 19.2% (48/250) were ineligible (Figure 1). The most common reason for ineligibility was not planning to exclusively breastfeed (n=31); just 3 individuals were ineligible because of not having a smartphone or an unlimited SMS plan. Participants were predominantly white, college educated, and married (Table 1).

**Table 1:** Demographics of study participants at baseline (13-25 gestational weeks; N=247).

Characteristic		MILK^a^ (n=124)	Text4Baby (n=123)	Total sample (n=247^b^)	*P* value^c^
Age (years), mean (SD)		28.9 (5.5)	28.7 (5.2)	28.8 (5.3)	.76
**Marital status, n (%)**					.81
	Married	81 (65.3)	77 (62.6)	158 (64.0)	
	Living with a partner	19 (15.3)	18 (14.6)	37 (15.0)	
	Single	24 (19.4)	28 (22.8)	52 (21.1)	
**Education, n (%)**					.81
	High school or less	15 (12.1)	13 (10.6)	28 (11.3)	
	Some college or vocational program	25 (20.2)	31 (25.2)	56 (22.7)	
	Bachelor’s degree	36 (29.0)	33 (26.8)	69 (27.9)	
	Postgraduate degree	48 (38.7)	46 (37.4)	94 (38.1)	
**Race,** **n (%)**					.64
	White	87 (70.2)	94 (76.4)	181 (73.3)	
	Black/African American	25 (20.2)	22 (17.9)	47 (19.0)	
	Asian/Indian	7 (5.6)	5 (4.1)	12 (4.9)	
	Mixed/biracial	3 (2.4)	2 (1.6)	5 (2.0)	
	Other	2 (1.6)	0 (0.0)	2 (1.0)	
Hispanic ethnicity, n (%)		3 (2.4)	7 (5.7)	10 (4.0)	.20
WIC^d^ recipient^e^, n (%)		32 (25.8)	28 (22.8)	60 (24.3)	.58
Employed, n (%)		104 (83.9)	108 (87.8)	212 (85.8)	.38
Smoke cigarettes (current and/or month before pregnancy), n (%)		14 (11.3)	16 (13.0)	30 (12.1)	.68
Prepregnancy BMI, mean (SD)		26.7 (7.1)	26.1 (5.5)	26.4 (6.3)	.43
Prepregnancy BMI ≥30 (obese), n (%)		24 (19.4)	24 (19.5)	48 (19.4)	.98
**Intended duration of any breastfeeding,** **n (%)**					.71
	<6 months	7 (5.6)	5 (4.1)	12 (4.9)	
	6-11 months	29 (23.4)	33 (26.8)	62 (25.1)	
	12 months	49 (39.5)	48 (39.0)	97 (39.3)	
	>12 months	18 (14.5)	12 (9.8)	30 (12.1)	
	Unsure/as long as possible	21 (16.9)	25 (20.3)	46 (18.6)	
**Intended duration of exclusive breastfeeding,** **n (%)**					.07
	<6 months	36 (29.0)	21 (17.1)	57 (23.1)	
	6 months or longer	51 (41.1)	64 (52.0)	115 (46.6)	
	Unsure/as long as possible	37 (29.8)	38 (30.9)	75 (30.4)	

^a^MILK is acronym for the intervention group treatment: a Mobile, semiautomated text message-–based Intervention to prevent perceived Low or insufficient milK supply.

^b^A total of three randomized participants did not complete the baseline demographic survey.

^c^*P* value for between-group differences were calculated with independent samples *t* tests for continuous type variables; for categorical variables, we used Pearson chi-square tests or, if sparse cells were encountered, Fisher exact tests.

^d^WIC: Special Supplemental Nutrition Program for Women, Infants, and Children.

^e^Of 246 participants answering the item.

### Uptake and Interactions with MILK (Usability)

In total, 38,284 MILK texts were sent to participants. Of those, 0.15% (58/38,284) texts sent to 38 participants were undelivered after multiple attempts. Nearly all MILK participants (122/126, 96.8%) engaged in at least one exchange with the system (eg, keyword text and response to query). Of 124 participants, 9 (7.3%) elected to stop MILK texts after receiving at least one message, and 3 (2.4%) participants elected to reduce messages to one series per week. In the control arm, 1 participant (1%) declined to receive any Text4Baby messages after randomization, and 11 participants (9%) were confirmed to have stopped Text4Baby messages (Figure 1).

There were 46 HELP requests from 25.4% (32/126) MILK participants for individualized assistance from the study’s IBCLC. Of these requests, 54% (25/46) occurred on a weekend, a holiday, or outside business hours (5 PM-7 AM). Most HELP requests specified SMS text messaging as the preferred medium to communicate with the study’s IBCLC (27/46, 59%). The most commonly texted keywords for more detailed information occurred during the postpartum period and focused on two issues: (1) assessing adequate breast milk volume/infant intake and (2) optimizing sleep within the breastfeeding relationship (Table 2).

**Table 2:** Most commonly texted MILK^a^ keywords.

Keyword	Timing of texts with a keyword prompt	Topical area	Number of participants texting, n
Sleep	Postpartum, weeks 6 and 7	Week 6: Maximizing sleep length/quality for the breastfed infant; Week 7: Tips for consolidating sleep for the breastfeeding mother	68; 54
Schedule	Postpartum, week 6	Norms for sleeping/feeding pattern emergence in infants; tips for encouraging the formation of desired sleeping/feeding habits	65
Norms	Postpartum, week 4	Average milk intake volume and weight gain for a 3- to 4-week-old infant	59
Reasons	Postpartum, week 5	Potential rationale for perceived decrease in milk volume between postpartum weeks 5 and 6	54
Sign	Postpartum, week 2	Indicators of satiety among breastfed infants	54

^a^MILK: Mobile, semiautomated text message-–based Intervention to prevent perceived Low or insufficient milK supply.

### Acceptability

At 8 weeks postpartum, 84% (82/98) of MILK participants completing a survey reported that MILK messages were “helpful” or “very helpful” in achieving their breastfeeding goals or helping them to breastfeed; 21% (18/88) in the control group reported the same for Text4Baby messages (between-group difference: χ^2^_1,186_=74.5; *P*<.001). According to the same survey, the most preferred MILK content was information about potential breastfeeding problems and solutions, while the least preferred was links to breastfeeding support resources (Table 3). The primary MILK dislike or criticism pertained to technical problems, though less than 10% (9/90) endorsed experiencing such issues (Table 4).

**Table 3:** Most preferred MILK^a^ text message content (n=101 MILK participants completing an 8-week survey). Participants could select more than one preferred message content type. No participants selected “other” or “did not like any messages.

Content type	Participants endorsing, n (%)
Encouragement to begin or continue breastfeeding	57 (56.4)
Information on how to prevent and manage breastfeeding problems	74 (73.3)
Information on breastfeeding recommendations	63 (62.4)
Information on breastfeeding benefits	42 (41.6)
Links to breastfeeding articles and websites	58 (57.4)
Links to videos featuring real parents breastfeeding	22 (21.8)
Links to connect with breastfeeding support groups or persons	14 (13.9)

^a^MILK: Mobile, semiautomated-automated text message–based Intervention to prevent perceived Low or insufficient milK supply.

**Table 4:** MILK^a^ text message dislikes and criticisms (n=101 MILK participants completing an 8-week survey). Participants could select more than one issue. No participants selected “content was hard to read/understand” [not represented in the table].

Dislike or criticism	Participants endorsing, n (%)
No dislikes	60 (59.4)
Too many texts	8 (7.9)
Too few texts	2 (2.0)
Messages too lengthy	1 (1.0)
Sent at an inconvenient time	7 (6.9)
Not helpful or applicable	4 (4.0)
Content offensive	3 (3.0)
Content poorly timed	5 (5.0)
Technical problems	9 (8.9)

^a^MILK: Mobile, semiautomated-automated text message–based Intervention to prevent perceived Low or insufficient milK supply.

### Qualitative Findings

A total of 35 MILK participants (28%) provided qualitative feedback on the intervention; 34 interviews were conducted at weeks 2 to 10 postpartum, and one was conducted at 6 months postpartum. We identified three major themes pertaining to participants’ experiences with MILK: (1) ascribed value rooted in the perceived impact on breastfeeding experience and trajectory; (2) preferred content prioritized practicalities, realities, and complexity in the breastfeeding relationship; and (3) appreciation for design features offering personalization and control juxtaposed with critiques about technical issues.

#### Theme 1: Perceived Impact on Breastfeeding Experience and Trajectory

Overwhelmingly, participants gave positive reviews of the MILK intervention, stating that it increased their breastfeeding knowledge and/or confidence, impacted their decision to begin or continue breastfeeding, and strengthened their breastfeeding support networks. In terms of knowledge/confidence, participants found MILK informative and reassuring in that the breastfeeding issues they were experiencing were surmountable and not uncommon:

I’m glad I signed up for the study, ‘cause I got like so much knowledge and was able to persevere through the tough times.

It gave me hope that other people had similar issues and made it through.

Participants stated that texts were often comprehensive enough that they did not need to consult with their pediatrician or use other resources (eg, books and the internet) for breastfeeding questions, though in-person counseling was still considered necessary to resolve complex breastfeeding issues. Participants also preferred MILK to other breastfeeding support resources for convenience and trustworthiness:

When I would get websites from you guys, I would know that this was a valuable resource, that it wasn’t just a random forum with somebody saying things, so I felt like it helped point you in the direction of the better information.

If I had certain kinds of [breastfeeding] questions, I’ll ask his doctor, but most of the time, the texts were just as helpful, so I didn’t have to bother them.

Participants noted that MILK texts reinforced their decision to breastfeed and provided encouragement to continue breastfeeding. In particular, texts were perceived as well-timed *(spot on; hit the nail right on the head*) to anticipate and address critical issues and misperceptions that might have otherwise led to formula supplementation or breastfeeding discontinuation:

I had almost given up breastfeeding even earlier, like after a week, and then one of the texts I got from the study was like, ‘This is the hardest part. This is a common growth spurt time, and it’ll get easier,’ and I kept going because of that.

A total of 1 participant noted that learning about the health impacts of breastfeeding through MILK provided a useful counternarrative to marketing tactics used by formula companies:

...they sent me Similac, some samples of it. And there are times when breastfeeding gets hard or it’s completely exhausting, and I thought, “Oh my gosh, it would be so easy just to give her formula right now.” But just knowing [from MILK] how beneficial it is for her to have purely breast milk was, I guess, was reinforcing of my decision

to exclusively breastfeed

Participants reported that particularly relevant or interesting texts were shared with partners or friends and “sparked” breastfeeding conversations. Texts were also thought to provide an advantage in breastfeeding discussions with pediatricians or IBCLCs:

They give me good terminology and language of how to talk about things...I was able to describe what was happening better because of some of the text messages.

#### Theme 2: Preferences for Content Encapsulating Breastfeeding Practicalities, Realities, and Complexity

Participants favored content sent in the postpartum period that provided anticipatory guidance about breastfeeding expectations at each stage of infant development (eg, first hour and first week), as well as when breastfeeding issues were most likely to surface, their causes, and how to avoid or resolve them. Participants particularly liked messaging that addressed breastfeeding frequency, how growth spurts impact breastfeeding, and indicators that the infant was getting enough breast milk. They reported often accessing video and weblinks from texts for more detailed information and “bookmarking” certain sites to return to later. Participants also found motivational messaging encouraging, particularly texts geared toward the achievement of incremental breastfeeding milestones:

And so when you’re looking at how long—“Oh my God, how long am I going to have to [breastfeed],” you can at least see those milestones, and feel like you’re accomplishing something when you hit, like, “Ok, like every single week that I do this, I’m helping my baby.”

In the antenatal period, participants noted that content addressing practicalities in preparation for breastfeeding was most helpful (eg, breast pumps and nursing clothing and bras). With several exceptions, antenatal messaging around the benefits of breastfeeding was deemed interesting but “too simplistic.” Participants felt that these texts could be replaced with information on how to practically integrate breastfeeding into the “real world.” In particular, they desired more guidance on breastfeeding in public spaces, pumping breast milk (eg, flange fit and recommended accessories), weaning and introduction of solid foods, formula supplementation, breastfeeding after primary teeth eruption, and resolving nipple soreness and plugged ducts:

Just tell me...like how to make [breastfeeding] work in my life, when I’m going to the mall.

Finally, some participants felt that several MILK messages “vilified” formula and that there was a general lack of messaging tailored for combination-feeding mothers. Conversely, some mothers thought texts were equally supportive of breastfeeding and formula feeding:

I felt like at the beginning, it was all about how breast milk was so much better than formula, and I definitely appreciate that breast milk has a lot of benefits, but at the same time, there’s, like, all sorts of reasons why people have to go on formula, you know?

You sent a website that said, about how moms like maybe only breastfeed at night and that still works, which I didn’t know that was a possibility. So it did help me to learn that there’s were like other options for me...and it kind of took away some of that mom guilt of “oh no, I’m giving my kid formula.”

Additional suggestions to improve MILK included tailoring content to address existing breastfeeding knowledge and more “complex” breastfeeding trajectories (eg, delayed lactogenesis and exclusively pumping), compiling all messages on a companion website for quick back-reference, adapting content for partners, and extending messaging beyond 8 weeks in recognition of continued breastfeeding barriers, such as the mother’s return to employment.

#### Theme 3: Appreciation for Design Features Offering Personalization and Control Juxtaposed With Critiques About Technical Issues

Participants liked receiving SMS text messaging–based breastfeeding support for various reasons, including convenience, autonomy in deciding when to read/access information, ability to refer back to texts, and “chunking” into a “consumable set of information”:

The information will be forever with me...I saved all my messages so I can go back and look at them...I would read them twice, maybe three or four times, just depending on how busy my day was.

Participants also responded positively to MILK’s automated personalization and interactivity features, including how they were addressed (“I loved how they used my baby’s name...it felt personal, even though I know that they’re automated text messages”) and the option to access more detailed information via keywords and links within messages. This interactivity seemed to contribute to some degree of anthropomorphism of the system, such that some participants began responding conversationally to automated messages. Participants also liked the ability to receive on-demand individual help from a study’s IBCLC. Those who solicited IBCLC assistance remarked that the consultant explained complex issues well, referred them to reputable resources, and provided “nonjudgmental,” personalized advice. Among those who did not use the IBCLC’s services, some had access to other lactation help, whereas others simply forgot and suggested weekly reminders.

Participants reported technical glitches in the system that somewhat diminished their enthusiasm for the program, including repeated audio alerts from multiple-part messages, broken weblinks and video links, and no response when texting a keyword (response windows “timed out” within 24 hours). To address these problems, multiple-part messages were consolidated where possible and weblinks were updated upon notification of an issue and during regular checks by the study staff. Similarly, we programmed an automated email alert for unsolicited texts. These alerts were reviewed daily to check for timed-out keyword texts, in which case the response was sent manually.

Opinions differed on delivery timing of automated messages. Although participants appreciated the predictability of a standard daily message time (10 AM) and felt that the message frequency was acceptable (no mention of preference for a particular day of the week), some reported that messages were disruptive during work or sleep. Some suggested early evening as a more convenient message delivery option, but there was no consensus.

## Discussion

We found that MILK was both a feasible and an acceptable breastfeeding support intervention among first-time mothers with a strong prenatal intention to breastfeed. During the trial period, we experienced rapid recruitment, a low rate of direct declines, and few withdrawals or elections to reduce message frequency. There was also a high rate of message receipt and participant interaction with the system. Participants reported that MILK increased their breastfeeding confidence, solidified their decision to initiate breastfeeding, and potentially helped them to breastfeed longer than they might have otherwise.

Other automated SMS breastfeeding support programs have been developed and trialed in China [22], Australia [21], Kenya [20], mainland United States [30], Hawaii, and Puerto Rico [31]. These systems all delivered approximately one automated text per week to breastfeeding mothers but varied in terms of initiation (pregnancy vs postpartum), duration (8 weeks to 12 months postpartum), personalization (eg, tailoring to key participant characteristics, such as breastfeeding status), access to an IBCLC, and rigor employed in content development. All but one program [31] reported positive results in terms of participant satisfaction and/or exclusive breastfeeding through 6 months postpartum. The MILK system differs from these existing programs in terms of its target population of first-time mothers, primary focus on a single breastfeeding issue (PIM), advanced automated functionality (eg, keyword texting and branched response logic), and grounding in both theory and EMA data [28]. The latter informed the delivery of near-daily texts tailored to breastfeeding issues experienced at the intersection of a particular gestational and chronological age. Although the impact of MILK on objectively measured breastfeeding outcomes are pending, the findings presented here illustrate a high degree of participant satisfaction with the overall program and its features. On the basis of suggested modifications to MILK, future SMS text messaging and technologically based breastfeeding support programs should consider messaging customized to one’s existing breastfeeding knowledge, current breastfeeding concerns, and unique breastfeeding trajectory. Such tailoring may be achieved via the incorporation of more advanced branching algorithms or possibly through the application of machine learning principles, though the user interface of any system should remain simple and intuitive [32].

A somewhat unexpected study finding was the relatively low utilization of MILK’s HELP feature providing consultation with an IBCLC. Although interview data indicate that participants may have simply forgotten about it, it is also possible that MILK prevented some common breastfeeding issues for which parents typically seek assistance. Another possibility, supported by the fact that more than half of the HELP requests came during off-hours, is that participants found community lactation resources more familiar or convenient. This is in line with the technology acceptance model, which states that for a technology to be adopted, the technology must fulfill both a need (perceived usefulness) and be more convenient than other options (perceived ease-of-use) [33]. Thus, future technologic breastfeeding innovations should consider what gap they fill relative to existing resources.

Our previous research indicated that among 146 surveyed postpartum mothers, the most commonly desired technology-based breastfeeding support involved encouragement or "cheerleading" [34]. However, mothers’ preferences for motivational breastfeeding support messages in this study was somewhat unanticipated, given that the automation of such content could be construed as generic or impersonal. Potentially, some of the personalization features of MILK, including addressing mothers and infants by name and referencing the infant’s age, contributed to a sense of connection to the system and openness to emotive appeals. This is supported by our observations that some participants began to actively converse with the system, even when a response was not indicated. Preferences for messages of encouragement also suggest that pregnant and postpartum individuals are not necessarily receiving this type of breastfeeding (or emotional) support within their existing networks [35]. Most participants also preferred practical how-to breastfeed messages rather than those addressing breastfeeding benefits and recommendations. It is unclear whether preferences would be similar among more diverse groups or among those with more ambivalent views toward breastfeeding.

The extrapolation of findings from MILK is limited by the sample demographics, consisting of predominantly white college-educated women without other biological children from a single geographic area and with a strong commitment to breastfeeding. In addition, MILK was designed for English speakers, though PIM is a pervasive lactation challenge across cultures [36-39]. Future research and programmatic development should consider SMS text messaging breastfeeding support for more diverse groups and for other common lactation challenges in addition to PIM.

## Appendix

Multimedia Appendix 1 CONSORT-EHEALTH checklist (V 1.6.1).

## Abbreviations

EMA: ecological momentary assessment
IBCLC: international board-certified lactation consultant
MILK: Mobile, semiautomated text message–based Intervention to prevent perceived Low or insufficient milK supply
PIM: perceived insufficient milk
